# Local mandate improves equity of paid sick leave coverage: Seattle’s experience

**DOI:** 10.1186/s12889-016-3925-9

**Published:** 2017-01-11

**Authors:** Jennifer L. Romich

**Affiliations:** University of Washington, Seattle, Washington USA

**Keywords:** Paid sick leave, Employer benefits, Local health mandate

## Abstract

**Background:**

Paid sick leave allows workers to take time off work for personal or family health needs, improving health and potentially limiting infectious diseases. The U.S. has no national sick leave mandate, and many American workers - particularly those at lower income levels - have no right to paid time off for their own or family members’ health needs. This article reports on outcomes of a local mandate, the City of Seattle Paid Sick and Safe Time Ordinance, which requires certain employers to provide paid sick leave to eligible workers.

**Methods:**

Survey collectors contacted a stratified random sample of Seattle employers before the Ordinance went into effect and one year later. Pre- and post- analysis draws on responses to survey items by 345 employers who were subject to the paid sick leave mandate.

**Results:**

Awareness of the policy and provision of paid leave grew significantly over the year after the Ordinance was enacted. More employers offered leave to full-time workers (80.8 to 93.9%, *p* < .001) and part-time workers (47.1 to 66.7%, *p* < .001) with particularly large increases in the hospitality sector, which includes food workers (coverage of any hospitality employee: 27.5 to 85.0%, *p* < .001).

**Conclusions:**

Absent a federal policy, local paid sick time mandates can increase paid sick leave coverage, an important social determinant of health.

## Background

Worldwide, workers in some 145 countries have protected paid sick time, which provides wages for missed work to employees who are absent due to temporary illness or incapacitation [1]. The U.S. lacks a national mandate, leaving 26% of full-time and 76% of part-time American private industry workers without paid leave [2]. Lower-wage, part-time and service sector workers are less likely than average to have paid leave [2]. Access to paid sick leave is associated with using preventative care including routine cancer screenings, [3, 4] parents’ ability to take care of sick children, [5, 6] and worker willingness and financial ability to avoid the workplace when ill [7, 8]. Expanding sick leave may reduce the spread of infectious diseases including influenza [9, 10].

Absent a national standard, 16 American cities or counties and five states have passed local mandates, extending paid leave to an estimated 11.3 million workers who would otherwise not have it [11]. While local paid sick leave ordinances are a separate area of policy from the federal health reform put in place by the 2010 Affordable Care Act (ACA), these state and local measures may complement the goals of the national policy. The lack of job protections and paid time off might limit use of new health coverage among workers hoping to avoid financial penalty for missed work. Hence for low-income populations, access to paid leave might increase the use of ACA-mandated health benefits [8].

Seattle, Washington numbered among early adopters of city mandates. The City of Seattle Paid Sick and Safe Time Ordinance (PSSTO) took effect September 1, 2012, requiring employers to provide paid leave to full-time, part-time, temporary, and occasional-basis workers who work within Seattle city limits [12]. PSSTO leave may be used for personal or family physical or mental health care needs; in the case of a workplace or child’s place of care being closed for public health reasons; or for reasons related to domestic violence, stalking or sexual assault. This article focuses on the first two, “sick time”, aspects. The Seattle Ordinance exempted small firms, those with four or fewer full-time-equivalent employees (FTEs) and established size tiers under which employees of larger employers have faster accrual rates and can use more time annually, up to a maximum of 72 h per year for most firms. Employees do not have to disclose reasons underlying the need for paid leave although employers can require documentation if the leave extends for more than three consecutive work days.

Understanding the potential public health and social equity impacts of local measures requires tracking whether such efforts make paid sick leave more available. Peer reviewed evidence about the effectiveness of local mandates is limited to an evaluation of the 2007 San Francisco mandate [13]. Before the San Francisco policy, 73% of firms offered paid sick leave; this rate increased to 91% post- ordinance [13]. The current study adds to knowledge about the effect of local mandates and makes two methodological advances. While the San Francisco findings rely on retrospective data, we measure within-firm changes over the implementation period. Our data differentiates between full- and part-time workers, better capturing impacts on the latter.

More broadly, this study offers evidence about the potential impact of policy reforms to expand paid sick leave and other labor standards, both at the local and national levels. City- and state-level health and labor standards such as the PSSTO are most likely in federalist systems such as the United States and Canada. Indeed, the strong American preference for local control and strength of the business sector rather than labor are offered as reasons for which the U.S. did not adopt federal paid leave standards at the same time many European nations did; European natalist policies and the need to encourage fertility are another explanation [14]. However, some European countries fund and implement social policy at local levels, per the subsidiarity principle which holds that needs should be addressed at the most local level possible. Although most countries world-wide offer paid leave to workers, far fewer (only 33 by one recent count) guarantee the right to use such time for the needs of children or other family members [1], suggesting that further expansion of paid leave could be considered in many countries. Additionally, guarantees of paid leave in developing nations with large informal workforces and scant resources for public enforcement mean that strengthening and enforcement of existing rules could constitute interventions similar in magnitude to the local ordinance examined in the current study.

## Methods

The aim of this study is to examine the outcomes of the PSSTO. Analysis focuses on two questions: do employers know about the Ordinance? and are they offering leave as required? Data draw from two rounds of surveys of Seattle employers conducted in July - October 2012 and August - November 2013, periods corresponding to the time the Ordinance took effect and one year later. Firms were recruited from a stratified random sample of Seattle Business License holders with industry oversamples in sectors with higher potential for infectious disease transmission: food service and accommodation (henceforth “hospitality”), retail trade, and health care and social services (henceforth “health care”).

An advance letter sent to *N* = 2319 employers asked the business “owner, manager or human resources director” to complete the survey. The letter stated the survey’s purpose as examining business practices in Seattle, including understanding the PSSTO, although this information was not repeated on the survey itself and PSSTO-specific questions specific were included last to avoid biasing responses to earlier items. The survey team mailed a paper survey and then contacted non-respondents by phone, achieving a baseline response rate of 63%. Of the baseline survey initial respondents, 551 indicated they had four or more employees and hence might be subject to the ordinance which applies to firms with more than 4.0 FTEs. We re-contacted these firms at follow-up via mail, phone and web, collecting responses from 79% of those attempted. Copies of the baseline and follow-up surveys are available as online supplements.

Data presented here are based on the sub-set of employers who responded to both waves and had five or more FTE employees, an analysis sample of *N* = 345 employers. Although the Ordinance applies to employers with “more than four” FTE, responses from interviewed employers suggested this cut-off was hard to understand [15]. Hence a more conservative estimate of firm eligibility based on five FTEs reduces the likelihood that some employers in the sample would be small enough to be exempt from the mandate. Responses are weighted to be representative of all Seattle employers subject to the ordinance. Weights reflect the within-strata likelihood of selection, the strata-specific estimate of the proportion of firms large enough to be affected by the Ordinance, and within-strata response rate. Analysis used the *svy:* function of Stata 14.

Employers who report offering paid sick time or general or universal paid time off (PTO) are coded as providing paid sick time. Employers who skipped or refused to respond to the paid sick time and PTO items were coded as not offering those benefits after an analysis of non-responses (*N* = 37, 22 in wave 1, 2) showed that the majority of these employers with missing data replied to other benefits questions, suggesting skipped items might tacitly indicate non-compliance.

## Results

Over two thirds of surveyed employers were aware of the PSSTO at the time it took effect, and awareness grew over the first year (Table 1) (69.1 to 83.5%, *p* < .001). At baseline, respondents at smaller employers (less than 50 FTE) were marginally less likely than those at larger employers to report knowing about the mandate (65.3 v. 77.4, *p* = .056); these two groups converged over the year as small employers became significantly more likely to affirm knowledge. Baseline knowledge was higher among the focal industries of hospitality, retail and health than among other employers (80.8 for focal v. 65.6 for other, *p* = .003), and awareness increased the most among non-focal industry employers.

**Table 1:** Employer Awareness of Paid Sick and Safe Time Ordinance, 2012 and 2013

Know about the Paid Sick and Safe Time Ordinance (%)				
	N	Baseline (2012)	1 year (2013)	
All employers	345	69.1	83.5	^***^
Size				
5-49FTE	247	65.3	83.1	^***^
50 + FTE	98	77.4	84.4	
Industry^a^				
Hospitality	40	82.5	82.5	
Retail	53	75.5	88.7	^**^
Health	61	86.9	93.4	
Other	191	65.6	82.4	^***^

SOURCE: Author analysis of data from survey of Seattle employers (see text)

^*^ *p* < .05, ^**^ *p* < .01, ^***^ *p* < .001

^a^Industries are classified using North American Industry Classification System (NAICS). Hospitality refers to firms with NAICS codes corresponding to Accommodation and Food Services Establishments. Health refers to firms with NAICS codes corresponding to Health Care and Social Assistance

At baseline, 79.5% of employers provided some paid sick to at least some employees (Table 2). Over the first year this rate increased to 90.6%. Employers with fewer than 50 employees were more likely than larger employers to add paid leave. The percentage of small employers providing leave increased from 75.2 to 89.4% (*p* < .001). Leave offering grew most in the hospitality industry, with the rate more than doubling from 27.5% when the Ordinance took effect to 85.0% one year later. Employers with full-time employees largely offered paid sick leave to full-time workers at baseline (80.8%) but this did grow significantly over the Ordinance’s first year (to 93.9%, *p* < .001). Smaller firms, hospitality firms, and those in the “other” sector showed significant increases in leave offering to full-time employees. In contrast to full-time employee offerings, fewer than half of employers offered leave to their part-time workers at baseline, but this increased to two thirds over the first year (47.1 to 66.7%, unweighted *p* < .001; weighted test with one small stratum dropped, *p* < =0.003). Both smaller and larger employers increased paid sick leave offering to part-timers, and the largest percentage increase was in the hospitality industry (3.0 to 69.7%, *p* < .001).

**Table 2:** Employer Provision of Paid Sick Time (PST), 2012 and 2013

		Provide PST to any employee (%)			N	Have full-time employees and provide PST to full-time employees (%)			N	Have part-time employees and provide PST to part-time employees (%)		
	N	Baseline (2012)	1 year (2013)			Baseline (2012)	1 year (2013)			Baseline (2012)	1 year (2013)	
All employers	315	79.5	90.6	^***^	268	80.8	93.9	^***^	163	47.1	66.7	^+++^
Size												
5-49FTE	230	75.2	89.4	^***^	198	77.5	93.4	^+++^	121	39.0	59.0	^++^
50 + FTE	85	89.3	93.5		70	88.7	95.1		42	65.9	84.3	^++^
Industry												
Hospitality	40	27.5	85.0	^***^	35	28.6	85.7	^***^	33	3.0	69.7	^***^
Retail Trade	50	78.0	88.0		41	82.9	95.1		27	44.4	70.4	^*^
Health	58	93.1	93.1		49	93.9	98.0		46	65.6	71.7	
Other	167	86.0	91.6	^*^	143	86.9	94.7	^**^	57	57.8	64.4	

SOURCE: Authors’ analysis of data from Survey of Seattle Employers (see text). Employers with employees covered by collective bargaining agreements (*N* = 30) not included, as bargaining units could waive PST mandate

^*^ *p* < .05, ^**^ *p* < .01, ^***^ *p* < .001 for adjusted Wald Test of weighted pre-post difference; ^++^ *p* < .01, ^+++^ *p* < .001 for F Test for unweighted pre-post difference (used when weighted subgroup results in stratum with single sampling unit)

Despite the significant increases in leave offering, some employers remain non-compliant, either not offering leave at all or not offering it to full- or part-time employees (more typically the latter). In total, 22.3% of employers reported not offering seemingly required paid sick leave. Analysis of other responses to the survey indicate that a third of these firms (34.4% of all non-compliers) reported not being aware of the Ordinance. Many (44.8%) believe their firms are compliant, perhaps not understanding that the mandate applies to part-time workers. Smaller numbers believe the ordinance does not apply to them, are aware that they are non-compliant, or do not know if they are compliant. Non-compliant employers are not significantly concentrated within any of the focal industries, but employers with fewer than 50 employees are more likely to be non-compliant (odds ratio .412, *p* < 0.063).

## Discussion

Employers grew more aware of Seattle’s sick leave mandate and more likely to offer paid sick leave over its first year of implementation. Overall 13.9% more employers offered leave one year post-PSSTO than at baseline, and the offering of leave to part-time workers increased more dramatically, with 41.6% more employers offering leave to part-timers in the follow-up.

In comparison to the San Francisco study which covered a longer period of time but relied on retrospective data, Seattle employers had higher baseline rates of leave offering but very similar overall coverage post-mandate (90.6% of Seattle employers and 91% of San Francisco employers provided paid sick leave) [13]. Although our study lacks a comparison group, the increase in paid sick leave offered in Seattle stands in contrast to national trends over the same time period which saw no change in the percentage of full-time private industry workers with leave (74%) and a slight increase in the percentage of part-time workers with leave from 24 to 25% [16, 17].

### Limitations

The study design necessarily yields limited information. First, the business respondents may have overstated leave offering in order to appear compliant. Although study materials clearly differentiated the study team from the City and assured participants about confidentiality, we cannot discern respondents’ truthfulness. Second, data only reflect half of the employer-employee relationship. The effectiveness of any paid sick leave mandate requires that workers know about and feel free to use paid leave. Our survey cannot capture informal practices that might discourage the use of paid leave. Finally, our baseline survey spanned the time when the Ordinance took effect on September 1, 2012. Paper survey copies were distributed before the Ordinance, but phone follow-up extended six weeks after. Hence baseline rates may reflect changes made in advance, underestimating the degree of change.

## Conclusions

Local paid sick leave mandates can dramatically increase the availability of paid sick leave, particularly for part-time and hospitality-sector workers who are at the greatest risk of not having leave in the general economy. When politics preclude federal advances in labor standards, mandates at the city level can reach substantial shares of U.S. workers given the concentration of economic activity in major cities [18].

Paid sick leave is an important social determinant of health in that it promotes health care use and reduces the workplace spread of infectious disease. Economically marginalized workers – those in lower-paying and part-time – positions are likely to gain access under mandated sick leave policies. For maximum public health impact, local mandates should be combined with strong enforcement, ensuring that workers both have access to leave and conditions that encourage its use.

## Abbreviations

FTE: Full time equivalent employee
PSSTO: City of seattle paid sick and safe time ordinance
